# Deepening Well-Being Evaluation with Different Data Sources: A Bayesian Networks Approach

**DOI:** 10.3390/ijerph18158110

**Published:** 2021-07-30

**Authors:** Federica Cugnata, Silvia Salini, Elena Siletti

**Affiliations:** 1 University Centre of Statistics for Biomedical Sciences (CUSSB), Vita-Salute San Raffaele University, 20132 Milano, Italy; cugnata.federica@hsr.it; 2 Department of Economics, Management and Quantitative Methods, Università degli Studi di Milano, 20122 Milano, Italy; 3 Department of Economic and Political Sciences, Università della Valle d’Aosta, 11020 Saint-Christophe, Italy; e.siletti@univda.it

**Keywords:** Bayesian networks, big data, well-being, life satisfaction, sentiment analysis (list three to ten)

## Abstract

In this paper, we focus on a Bayesian network s approach to combine traditional survey and social network data and official statistics to evaluate well-being. Bayesian networks permit the use of data with different geographical levels (provincial and regional) and time frequencies (daily, quarterly, and annual). The aim of this study was twofold: to describe the relationship between survey and social network data and to investigate the link between social network data and official statistics. Particularly, we focused on whether the big data anticipate the information provided by the official statistics. The applications, referring to Italy from 2012 to 2017, were performed using ISTAT’s survey data, some variables related to the considered time period or geographical levels, a composite index of well-being obtained by Twitter data, and official statistics that summarize the labor market.

## 1. Introduction and Background

In recent decades, since the Stiglitz Commission’s suggestions advising to build a complementary system focused on social well-being that is suitable for measuring sustainability and that considers subjective assessment [1], different new measures of well-being have been proposed. In these new indices, the subjective dimensions are traditionally investigated using ad hoc surveys, but these data are not free of issues. Some issues are related to the survey structure or plan, which, despite all efforts [2], still create some methodological drawbacks [3,4]; other issues are related to the poor geographical disaggregation or low time frequency of the data.

An objective evaluation of the currently proposed indices demonstrates a limited and undersized presence of data in the the subjective and perceived dimension. Since 2012, with the aim of finding complementary information to fill this information gap, a new subjective and perceived Italian well-being index from social networks data has been proposed (Subjective Well-Being Index (SWBI) [5]). This is a composite index that uses the same framework considered by the New Economic Foundation for its Happy Planet Index [6], but it is the result of a human-supervised sentiment analysis (integrated sentiment analysis (iSA) [7]) of Twitter data. Recently, suggested using the SWBI to provide information on subjective well-being at Italian sub-national levels and at different moments in time [8].

Despite social network data being defined as the largest available focus group in the world [9,10], as they cover several topics, are continuously updated, and are free or cheap, these data also have disadvantages. Even if social media users are always increasing in number (from http://wearesocial.com 27 January 2021: from January 2019 to January 2020 the world growth was +7% in Internet access (59% of people in the world had Internet access) and +9.2% for active social media accounts (with a penetration of 49%)), not all are users; hence, one of the main issues with this kind of information is sampling bias. To enable the use of such a rich source of information, scholars are still working on the solution to these issues. Notably, suggested a new Italian well-being measure combining official statistics with Twitter data using a weighting procedure combined with a small area estimation (SAE) model to precisely consider sampling bias [11].

For a detailed description of all the well-being index in Italy, please refer to the work of [12]. This paper focuses on the Italian scenario due to the central role given to this topic by the Italian Parliament, which introduces equitable and sustainable well-being among the objectives of the government’s economic and social policy. The authors provided a detailed outline of the well-being indices useful for different scholars and practitioners, with the awareness that, for a good analysis, a complete and conscious description of disposable data is the starting point to further improve their usefulness, maximize their advantages, and reduce their limitations.

In this study, using a a Bayesian network (BN) approach, we combined social networks data, which are characterized by high frequency and geographical disaggregation, traditional survey data, and official statistics to evaluate well-being. Adopting this approach, both categorical and continuous data can be used with different geographical area levels and time frequencies. The aims of this study were twofold: (1) to describe the relationship between survey and social network data; (2) to investigate social network data and official statistics. We focused on the forecasting power of the social media information. All analyses were performed using R version 4.0.4 (R Core Team (2020). R: A language and environment for statistical computing. R Foundation for Statistical Computing, Vienna, Austria. URL: https://www.R-project.org/, downloaded 30 June 2021). Section 2.1 reports a brief presentation of BNs. Section 2.2 describes the data. Section 3 provides a discussion of the results.

## 2. Materials and Methods

### 2.1. Bayesian Networks: A Short Refresher

A Bayesian network (BN) is a probabilistic graph model that represents a set of stochastic variables with their conditional dependencies through the use of a direct acyclic graph (DAG). For example, a Bayesian network could represent the probabilistic relationship existing between symptoms and diseases. Given the symptoms, the network can be used to calculate the probability of the presence of different diseases.

Formally, Bayesian networks are direct acyclic graphs whose nodes represent random variables in the Bayesian sense: they can be observable quantities, latent variables, unknown parameters, or hypotheses. The arcs represent conditions of dependence; the nodes that are not connected represent variables that are conditionally independent of each other. Each node is associated with a probability function which takes as input a particular set of values for the variables of the parent node and returns the probability of the variable represented by the node. For example, if the parents of the node are Boolean variables, then the probability function can be represented by a table in which each entry represents a possible combination of true or false values that its parents can assume. There are efficient algorithms that perform inference and learning starting from Bayesian networks.

BNs are both mathematically rigorous and intuitively understandable data analytic tools. They implement a graphical model structure that is popular in statistics, machine learning, and artificial intelligence. They enable the effective representation and computation of a joint probability distribution (JPD) over a set of random variables.

The DAG’s structure is defined by a set of nodes, representing random variables and plotted by labeled circles, and a set of arcs, representing direct dependencies among the variables and plotted by arrows. Thus, an arrow from $X_{i}$ to $X_{j}$ indicates that a value taken by variable $X_{j}$ depends on the value taken by variable $X_{i}$. Node $X_{i}$ is referred to as a “parent” of $X_{j}$. Similarly, $X_{j}$ is referred to as a “child” of $X_{i}$. An extension of these genealogical terms is often used to define sets of “descendants”, i.e., the set of nodes from which the node can be reached on a direct path. The DAG guarantees that there is no node that can be its own ancestor (parent) or its own descendent. This condition is of vital importance to the factorization of the joint probability of a collection of nodes. Although the arrows represent direct causal connections between the variables, under the causal Markov condition, the reasoning process can operate on a BN by propagating information in any direction. A BN reflects a simple conditional independence statement, namely that each variable, given the state of its parents, is independent of its non-descendants in the graph. This property is used to reduce, sometimes significantly, the number of parameters that are required to characterize the JPD. This reduction provides an efficient method to compute the posterior probabilities given the evidence in the data [13,14]. In addition to the DAG structure, which is often considered to be the qualitative part of the model, the quantitative parameters are estimated by applying the Markov property, where the conditional probability distribution at each node depends only on its parents.

More formally, BNs are defined by a network structure: a DAG G=(V;A), in which each node $v_{i}\in V$ corresponds to a random variable $X_{i}$, and a global probability distribution X, which can be factorized into smaller local probability distributions according to the arcs $a_{ij}\in A$ in the graph. The main role of the network structure is to express the conditional independence relationships among the variables in the model through graphical separation, thus specifying the factorization of the global distribution:

$$\prod_{i=1}^{p}P(X_{i}|\Pi_{X_{i}})\;\;\;\;\text{where}\;{\;}_{X_{i}}=\text{parents of}\;X_{i}.$$

The probability distribution P(X) should be chosen such that the BN can be learned efficiently from the data. This distribution is flexible, so the assumptions should not be too strict, and it is easy to perform inference query [15]. The three most common choices in the literature are:

- Discrete BNs (DBNs): *X* and $X_{i}|\Pi_{X_{i}}$ are multinomial.
- Gaussian BNs (GBNs): *X* is multivariate normal and $X_{i}|\Pi_{X_{i}}$ are normal.
- Conditional linear Gaussian BNs (CLGBNs): *X* is a mixture of multivariate normal and $X_{i}|\Pi_{X_{i}}$ are multinomial, normal, or mixtures of normal.

It was proved that exact inference is possible in these three cases, hence their popularity. In this study, we considered both CLGBNs and GBNs. In the first aim of this paper (Section 3.1), some variables are categorical and others are numerical; instead, for the second aim (Section 3.2), all the variables are numerical.

To compare the strength of the link between two variables, we evaluated the strength as measured by the score gain/loss that would be caused by the arc’s removal [16]. Especially, we adopted the BIC criterion. The strength is the difference between the score of the network in which the arc is not present and the score of the network in which the arc is present.

BNs have been used in the analysis of multi-dimensional well-being [17], considering the correlation among dimensions. The same authors also applied multivariate statistical techniques to ISTAT’s BES index [18].

Moreover, employed BNs in the context of subjective well-being, focusing on the ability to predict it using material living conditions and deprivation, using the European Quality of Life Study data (2011) in four Central European countries. Our proposed method combines, in a BNs approach, well-being data from social networks [5] and surveys [19].

### 2.2. The Data

In this study, using a Bayesian networks (BNs) approach, we combined traditional survey, social networks data, and official statistics to evaluate well-being. In the first step, we described the relationship between survey and social network data. In the second step, we investigated social network data and official statistics. In this section, we introduce the different kind of used data.

As survey data, we considered some variables from the Aspects of Daily Life report. This ISTAT sample survey collects fundamental details on individual and household daily Italian life, focusing on several thematic areas of different social aspects useful to studying well-being. These data are also adopted for the subjective dimension in the ISTAT’s well-being index Benessere Equo e Sostenibile (Equitable and Sustainable Well-Being in Italian, BES). This is an annual survey that considers people aged 14 years and over, and the data, with a regional aggregation, are available free of charge. Data download was done on 30 June 2021. (http://dati.istat.it/).

The considered variable were:

- I_sat is the average rating of satisfaction with life as a whole (on a 1 to 10 scale).
- I_eco_sat is the percentage of people very or fairly satisfied with their economic situation.
- I_health_sat is the percentage of people very or fairly satisfied with their health.
- I_family_sat is the percentage of people very or fairly satisfied with family relationships.
- I_friends_sat is the percentage of people very or fairly satisfied with their friendships.
- I_freetime_sat is the percentage of people very or fairly satisfied with their free time.

In the first step, we also considered some variables related to the considered time period and geographical area:

- w_day is the day of the week.
- month is the month.
- year is the year.
- prov is the Italian province.

**Figure 1:**
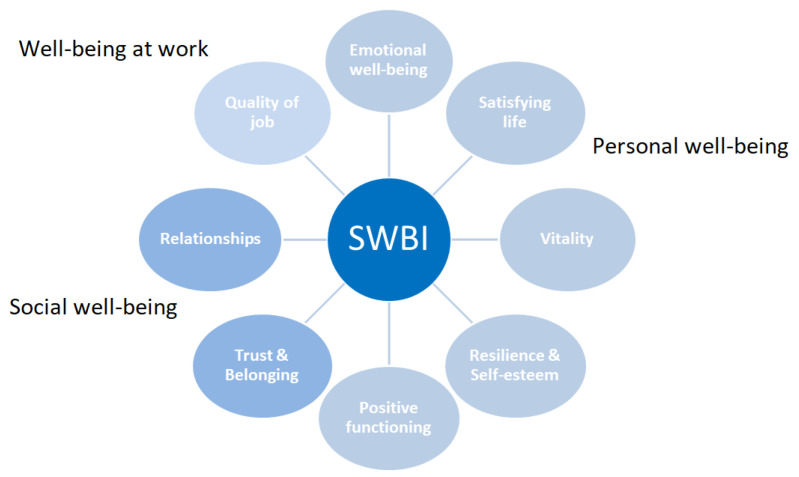
The SWBI framework. The three different shades of blue are defined according to the dimension of well-being.

Focusing on social network data, we used the SWBI index [8], which is defined by eight domains, all measured on a 0–100 scale, according to three well-being dimensions: *personal well-being*, *social well-being*, and *well-being at work*. In Figure 1, the eight domains are characterized by three different shades of blue according to the dimension of interest: *personal well-being* summarizes the person’s self-perception and is defined across five different domain; *social well-being* concerns perceptions of the individual’s relations with other people and is composed of two domains; “well-being at work” is represented by the subjective assessment of one’s own as far as work is concerned and refers to only one domain. This framework was inspired by the Happy Planet Index (HPI) of the New Economy Foundation (NEF) [6], and, during the coding definition of the human supervised step of the iSA-integrated Sentiment Analysis [7] process, the sentences were coded considering this structure.

Sentiment analysis is a methodology used for the systematic extraction of web users’ emotional states from the texts that they post on various platforms, such as blogs, social networks, etc. The literature in social psychology highlights an association between the well-being of individuals and their use of words. This implies that it is possible to extract words from the messages posted on social networks to reconstruct the emotional content, infer psychological traits, and measure the subjective well-being of individuals [20].

In the iSA algorithm, the human supervised part is essential because information can be retrieved from texts without relying on dictionaries of special semantic rules. Humans just read a text and associate a topic (e.g., life satisfaction) with it. Then, the computer learns the association between the set of words used to express that particular opinion and extends the same rule to the rest of the texts.

Human coders classify a small percentage of the texts (*training set*), in order to train the computer program to associate specific words with the dimensions of well-being described above. Then, all remaining data are automatically classified (*test set*). Each tweet is classified according to the scale −1, 0, 1, where −1 is negative, 0 is neutral, and 1 is positive feeling. The data used for the empirical estimation of the SWBI are represented by tweets written in Italian and posted in Italy. A percentage of tweets contains geo-reference information, which makes it possible to build indicators with a high geographical disaggregation. In the considered dataset, more than 200 million tweets were analysed. They were collected with daily frequency and for all Italian provinces. For an up to date and complete comprehension of the SWBI index and, in general, of the social media data, we suggest the work of [21]. In this paper, we only consider the following domains:

- sat: Life satisfaction, having positive assessment of the overall life situation.
- vit: Vitality, having energy, feeling well-rested and healthy, and being active.
- rel: Relationships, the degree and quality of interactions in close relationships with family, friends, and others who provide support.
- wor: Job quality, feeling satisfied with employment, work–life balance, and evaluating the emotional experiences of work and work conditions.

In the second step of our analysis, we used one official statistic that is traditionally used to evaluate the labor world: the quarterly regional unemployment rate (t_unemployment).

As in the used data there are different time frequencies, and SWBI has higher-frequency data (daily), all data were integrated at the daily level using repeated values for annual and quarterly data.

## 3. Empirical Results and Analysis

### 3.1. Social Media Data vs. Survey Data

The first step of our analysis was aimed to understand if a coherence exists among subjective measures of well-being; the high-time-frequency social network data, composed of a structured and and emotional component [7]; the annual survey data from the Aspects of Daily Life report (ISTAT). The analysis was performed considering six survey variables (I_sat, I_eco_sat, I_health_sat, I_family_sat, I_friends_sat, and I_freetime_sat), four SWBI variables (sat, vit, wor, and rel), and four covariates (year, month, day of week, and province). The analysis was performed using R statistical language, using the bnlearn [15] and BNViewer libraries [22].

**Figure 2:**
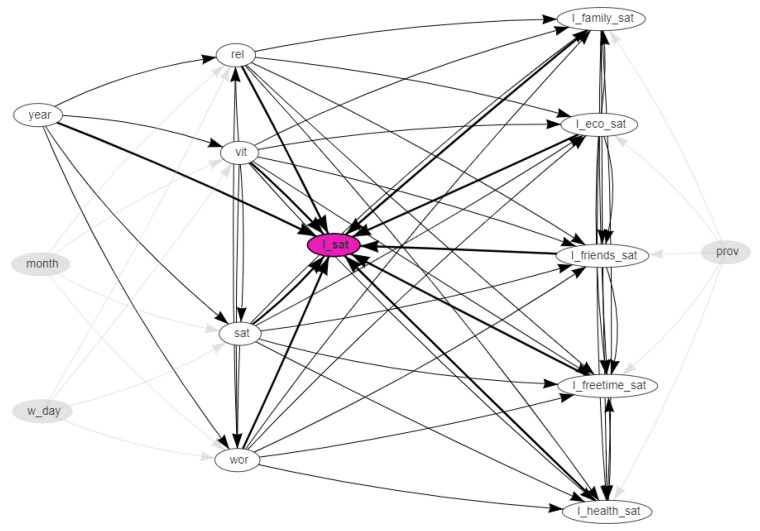
Social media data vs. survey data: The Bayesian network. The parent nodes of the ISTAT’s overall satisfaction node (I_sat), in purple, are highlighted in white. The directed edges (arcs) model the probabilistic dependencies between the different nodes.

**Figure 3:**
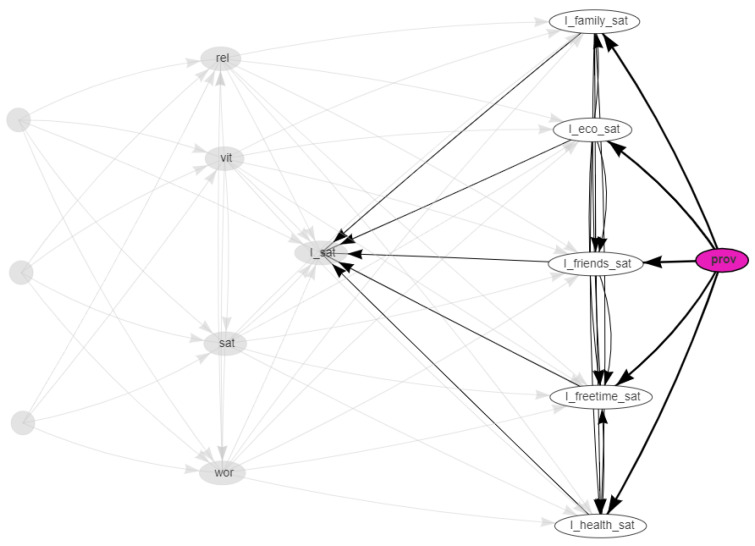
Social media data vs. survey data: Bayesian network highlighting the Italian provinces (purple). The children nodes of the province nodes are in white.

**Figure 4:**
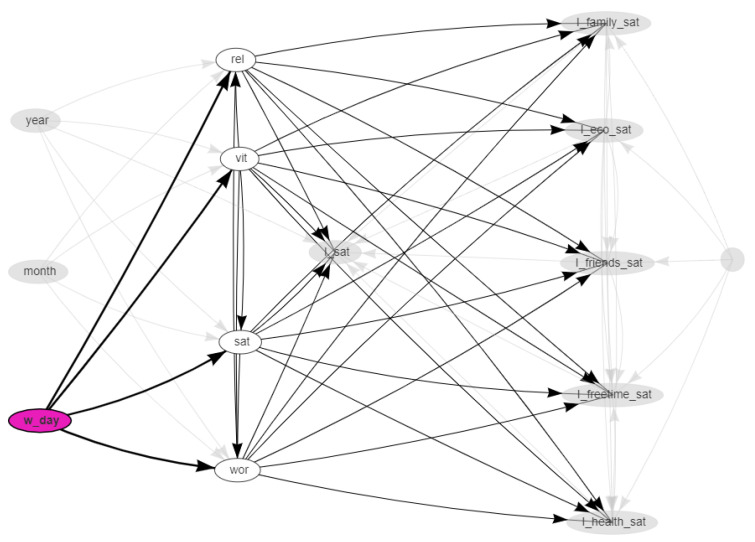
Social media data vs. survey data: Bayesian network highlighting the day of the week. The children nodes of the day nodes are in white.

The network was obtained with the hill-climbing algorithm with the BIC-CG score functions. The resulting network is shown in Figure 2, Figure 3 and Figure 4. In these dynamical plots, the highlighted nodes are in purple, while the children or parents of the highlighted nodes are in white.

Figure 2 highlights the parents nodes of ISTAT’s overall satisfaction in white. The ascendents that are not parents are in grey. The structure of the our obtained network allows us to respond with the affirmative to our initial research question: the ISTAT’s overall satisfaction is linked both to the individual dimensions of the survey and to the variables that comprise the social SWBI.

Figure 3 highlights the descendent nodes of the provinces node, the children are denoted by white nodes. It is evident that the geography dimension has an impact on the measurements obtained through the surveys, namely direct with the single dimensions and mediated by the global index of satisfaction (I_sat), but its effect on social data, characterized by wider variability due to the emotional component, is negligible. This result is in line with those reported in [8].

Figure 4 highlights the descendent nodes of the week day. As expected, the day of the week has a direct impact on the indices obtained by Twitter data, sat, vit, wor, and rel, but it has no effect on the ISTAT’s satisfaction indices, which are annual. The same was observed for the month, as can be found by examining the arrows starting at months.

### 3.2. Social Media Data vs. Official Statistics

In the second step, we investigated social network data and official statistics. We focused on the forecasting power of social media information.

We used an official statistic that is traditionally used to evaluate the labor world: the quarterly regional unemployment rate (t_unemployment).

We aimed to determine if the social variables, in particular the wor variable, job quality, is able to anticipate the information in the official measure of the unemployment rate, which refers to previous months.

**Figure 5:**
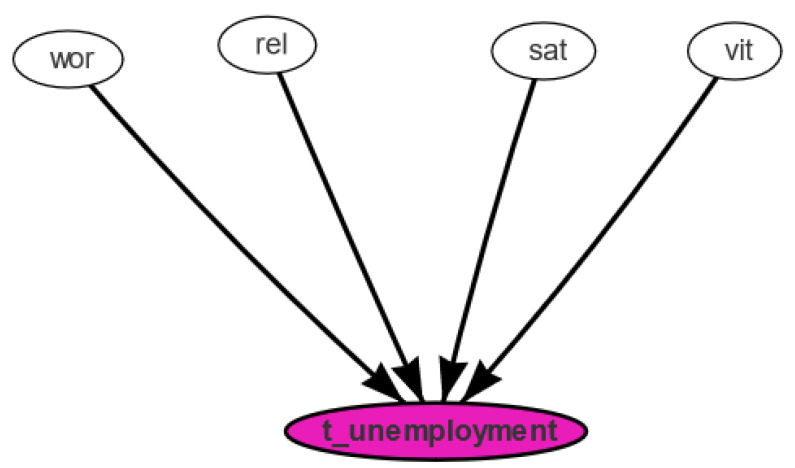
Social media data vs. official statistics: Bayesian network with the social network domains and the unemployment rate with a 90-day delay.

For this purpose, we tried to evaluate the effect of wor using the strength of the link to the unemployment rate, with lags of 90, 120, 270, and 360 days. Figure 5 reports the Bayesian network obtained with the hill-climbing algorithm with the BIC score functions, with a 90-day lag. As such, we confirmed that all the social dominions are related to the official statistics.

For completeness, we stress that, when we considered the other lags (120, 270, and 360 days), similar networks were obtained: all the links were confirmed and the networks differed only in the strength of the correspondence with the different arcs.

**Figure 6:**
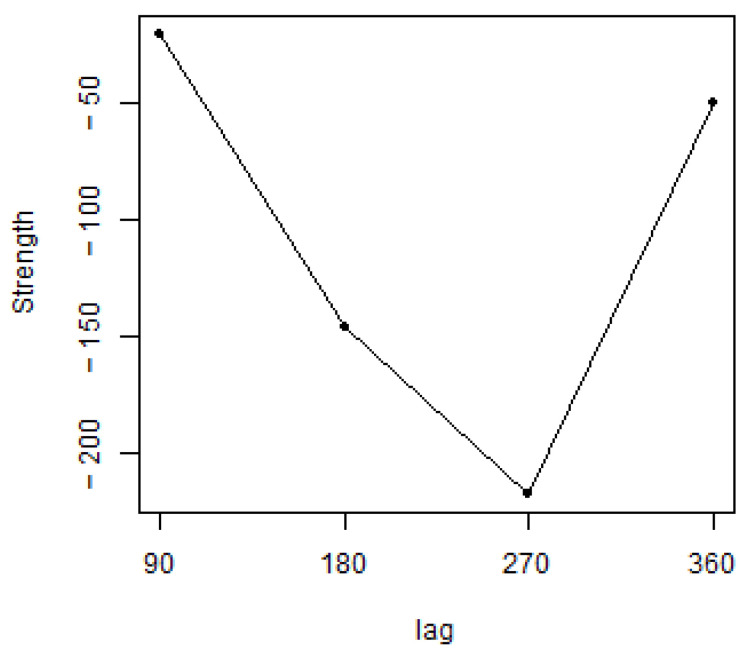
Social media data vs. official statistics: strength of the arc from the quality of job (wor) and the unemployment rate with different data lags.

The strength is measured by the score gain/loss that would be caused by the arc’s removal [16]. In other words, it is the difference between the score of the network in which the arc is not present and the score of the network in which the arc is present. Negative values, represented on the vertical axis in Figure 6, correspond to decreases in the network score; positive values correspond to increases in the network score (the stronger is the relationship, the more negative is the difference).

Considering the lags of 90, 180, and 270 days, we found an increasingly stronger relationship between the social information (wor) and the official statistic (t_unemployment). When considering the 360-day lag, corresponding to an annual delay, the forecasting power of the SWBI decreases, but the difference is still negative.

## 4. Conclusions

Evaluating well-being has involved the work of many scholars, and new methods have recently characterized the international scientific research in this field. In the European context, the work of the Stiglitz Commission [1] represents an epochal turning point, among others.

Nowadays, well-being measures must involve both objective and subjective components, provide comparable indicators, have good territorial granularity within the nation, and be up to date. Another aspect highlighted by [23] is the need to integrate different data: by source, by representativeness, by time frequency, and by territorial granularity. In this method, we incorporated all these aspects: applying Bayesian networks, we defined two questions for which we arrived at an affirmative answer for both.

In Section 3.1, we describe the relationship between the survey and social network data. Because the relationships among survey and social network data are complex, the BNs approach was used to evaluate the depending structure.

The network that we obtained allowed us to answer “yes” to our first research question: the ISTAT’s survey data are linked to the variables that comprise the social SWBI. The roles of the different covariates are confirmed.

In Section 3.2, we investigate social network data and official statistics, with a particular focus on the forecasting power of social media information. We confirmed that the social variables, in particular job quality (wor), predicts the information of the official measure of the unemployment rate. With a 270-day delay, i.e., nine months, we identified a stronger relationship.

In the future, we will focus on developing the analysis to evaluate if social network data, focusing on other domains, are able to anticipate other official statistics. This study will be important because the availability of official statistics with a reasonable time frequency and a realistic geographical granularity is limited.

## Data Availability

The SWBI replication R scripts and data will come with the upcoming book *Subjective Well-Being and Social Media* by Stefano M. Iacus, Giuseppe Porro, by Chapman and Hall/CRC.

## References

[1] Stiglitz J., Sen A., Fitoussi J.P. Report by the Commission on the Measurement of Economic Performance and Social Progress. INSEE, 2009. https://ec.europa.eu/eurostat/documents/8131721/8131772/Stiglitz-Sen-Fitoussi-Commission-report.pdf.

[2] Kahneman D., Krueger A.B. (2006). Developments in the measurement of subjective well-being. J. Econ. Perspect..

[3] Deaton A. (2012). The Financial Crisis and the Well-Being of America.

[4] Feddersen J., Metcalfe R., Wooden M. (2016). Subjective wellbeing: Why weather matters. J. R. Stat. Soc. A Stat..

[5] Iacus S.M., Porro G., Salini S., Siletti E. (2015). Social networks, happiness and health: From sentiment analysis to a multidimensional indicator of subjective well-being. arXiv.

[6] NEF (2012). The Happy Planet Index: 2012 Report. A Global Index of Sustainable Well-Being.

[7] Ceron A., Curini L., Iacus S.M. (2016). iSA: A fast, scalable and accurate algorithm for sentiment analysis of social media content. Inf. Sci..

[8] Iacus M.S., Porro G., Salini S., Siletti E. (2019). An Innovative Measurement for Italian Provinces. Ital. J. Reg. Stu..

[9] Hofacker C.F., Malthouse E.C., Sultan F. (2016). Big Data and consumer behavior: Imminent opportunities. Ital. J. Consum. Mark..

[10] Kwong B.M., McPherson S.M., Shibata J.F.A., Zee O.T. (2012). Facebook: Data mining the world’s largest focus group. Graziadia Bus. Rev..

[11] Iacus S.M., Porro G., Salini S., Siletti E. (2020). Controlling for Selection Bias in Social Media Indicators through Official Statistics: A Proposal. J. Off. Stat..

[12] Facchinetti S., Siletti E. (2021). Well-being Indicators: A Review and Comparison in the Context of Italy. Soc. Indic. Res..

[13] Lauritzen S.L., Spiegelhalter D.J. (1988). Local computations with probabilities on graphical structures and their application to expert systems. J. R. Stat. Soc. B.

[14] Pearl J. (2000). Causality: Models, Reasoning, and Inference.

[15] Scutari M. (2010). Learning Bayesian networks with the bnlearn R package. J. Stat. Softw..

[16] Scutari M., Nagarajan R. On Identifying Significant Edges in Graphical Models. Proceedings of the Workshop ‘Probabilistic Problem Solving in Biomedicine’ of the 13th Artificial Intelligence in Medicine Conference.

[17] Ceriani L., Gigliarano C. (2016). Multidimensional well-being: A Bayesian Networks approach. ECINE Society for the Study of Economic Inequality.

[18] Chelli F.M., Ciommi M., Emili A., Gigliarano C., Taralli S. (2016). Assessing the Equitable and Sustainable Well-Being of the Italian Provinces. Int. J. Uncertain Fuzz..

[19] Svorc J., Vomlel J. Employing Bayesian Networks for Subjective Well-being Prediction. Proceedings of the 11th WUPES’18.

[20] Quercia D., Mejova Y., Weber I., Macy M.W. (2015). Hyperlocal Happiness from Tweets. Twitter: A Digital Socioscope.

[21] Iacus S.M., Porro G. (2021). Subjective Well-Being and Social Media.

[22] Fernandes R. Bnviewer: Interactive Visualization of Bayesian Networks. R Package Version 0.1.4. https://CRAN.R-project.org/package=bnviewer.

[23] Iacus S.M., Porro G., Salini S., Siletti E. (2020). An Italian Subjective Well-being Index: The Voice of Twitter Users from 2012 to 2017. Soc. Indic. Res..

